# Interspinous Bursitis due to *Staphylococcus aureus* in an Adolescent

**DOI:** 10.1155/crpe/7740167

**Published:** 2026-09-16

**Authors:** Florine Foré, Yasmine Lamrous, Antoine Delval, Audrey Vanrenterghem, Djamal Djeddi

**Affiliations:** ^1^ Department of Pediatrics, Amiens-Picardie University Hospital, Amiens, France; ^2^ Pediatric Radiology Unit, Amiens-Picardie University Hospital, Amiens, France

**Keywords:** adolescent, interspinous bursitis, lower back pain, MRI diagnosis, *Staphylococcus aureus*

## Abstract

**Background:**

Interspinous bursitis is exceptionally rare in children, contrasting with degenerative forms in adults.

**Case Presentation:**

A 16‐year‐old male presented with acute severe low back pain, fever (39°C), and focal L3‐L5 tenderness. CRP was 233 mg/L; blood cultures grew methicillin‐susceptible *Staphylococcus aureus*. Imaging progression (US, CT‐scan, MRI, PET‐CT) confirmed L3‐L4 interspinous bursitis without bone/disc involvement, distinguishing it from spondylodiscitis.

**Management:**

Cefazolin IV (10 days) followed by oral ciprofloxacin (4 weeks), a lumbar brace, and rehabilitation yielded complete recovery.

**Conclusions:**

Pediatric interspinous bursitis presents as febrile back pain via hematogenous dissemination. MRI is diagnostic, excluding spondylodiscitis. Conservative management with early antibiotic switch is effective when imaging excludes bone/disc involvement. This case highlights distinguishing features and optimal management of this rare pediatric entity.

## 1. Introduction

Interspinous bursitis is an inflammatory process that affects the bursal sac situated between the lumbar spinous apophyses—most frequently in the region between the third and fifth lumbar vertebrae (L3 and L5). In older adults, the inflammation is often related to degenerative or mechanical changes. Most of the rare cases reported in children and adolescents involve *Staphylococcus aureus* via dissemination in the blood from a skin lesion or without a clearly identified entry point [1].

## 2. Case Report

A 16‐year‐old male with an unremarkable medical history presented with acute, severe lower back pain that had started 48 h earlier and prevented him from sitting down. He also presented with fever (a temperature of up to 39°C) and poor general condition but no hemodynamic instability. A clinical examination revealed focal, lumbar tenderness and subcutaneous swelling over the L3–L5, in the absence of nerve root disorders or neurological impairments. No local or remote skin damage was found. The serum C‐reactive protein (CRP) level was elevated (233 mg/L), but there was no leukocytosis (leukocyte count: 9.7 × 10^9^/L; neutrophils: 70%). Three independent sets of blood cultures were positive for *Staphylococcus aureus* (MALDI‐TOF mass spectrometry). The methicillin‐susceptible (MS) phenotype was confirmed by standard testing. A specific minimum inhibitory concentration (MIC) value was not provided by our routine laboratory report, which relies on qualitative categorizations (S/I/R). A mecA PCR was not performed, as the phenotypic testing clearly identified an oxacillin‐susceptible isolate, which is standard practice in our institution for unambiguous MSSA strains. The susceptibility profile revealed an inducible MLSb phenotype (resistant to erythromycin, susceptible to clindamycin, with a positive D‐test inferred). The isolate also showed susceptibility to levofloxacin, aminoglycosides, linezolid, rifampicin, and doxycycline.

**FIGURE 1:**
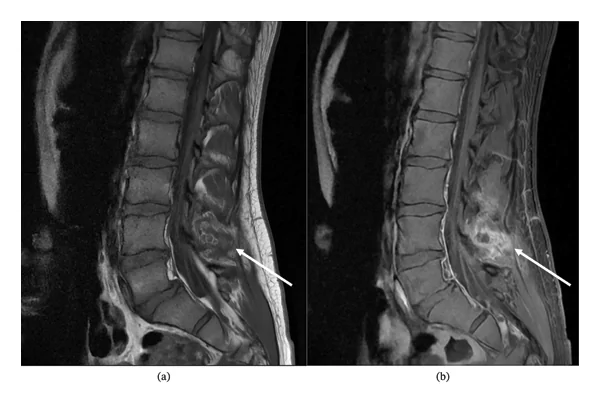
(a) Sagittal MRI of the lumbar region (a gadolinium contrast‐enhanced T1 sequence with fat saturation). The white arrow indicates peripheral enhancement of the bursa, associated with inflammation of the interspinous bursa between the third and fourth lumbar vertebrae (L3 and L4). There was no disc or bone involvement. (b) Sagittal MRI of the lumbar region (a gadolinium contrast‐enhanced T1 sequence with fat saturation). The white arrow indicates peripheral enhancement of the bursa, associated with inflammation of the interspinous bursa between the third and fourth lumbar vertebrae (L3 and L4). There was no disc or bone involvement.

**FIGURE 2:**
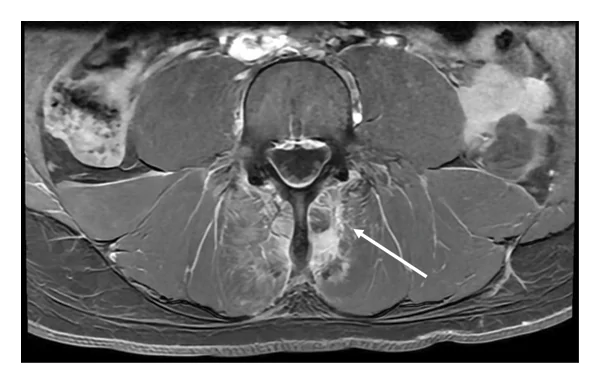
Transverse MRI of the lumbar region (a gadolinium contrast‐enhanced T1 sequence with fat saturation). The white arrow indicates peripheral enhancement of the bursa, associated with inflammation of the interspinous bursa between the third and fourth lumbar vertebrae (L3 and L4). There was no disc or bone involvement.

**FIGURE 3:**
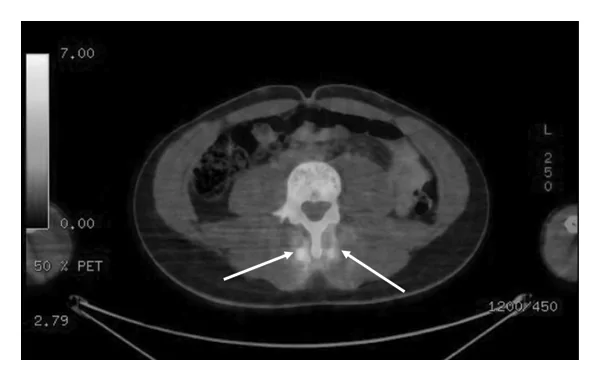
Transverse fluorodeoxyglucose positron emission tomography‐CT (PET‐CT) imaging of the lumbar region. The white arrow indicates peripheral enhancement of the bursa, associated with inflammation of the interspinous bursa between the third and fourth lumbar vertebrae (L3 and L4).

A soft‐tissue ultrasound scan revealed subcutaneous infiltration but no fluid collection. Lumbar CT revealed interspinous bursitis at L3‐L4, together with infiltration of the adjacent paravertebral muscles. The presence of an interspinous collection was shown on MRI, with hypointensity on T1‐weighted sequences and hyperintensity on T2‐weighted sequences but no associated disc or bone involvement (Figures 1a,b and 2). Fluorodeoxyglucose positron emission tomography (PET) CT showed moderate hypermetabolism of the paravertebral muscles at L4/L5, with no evidence of another infection site (Figure 3). The transthoracic echocardiographic findings were normal.

Intravenous (IVs) antibiotic therapy with cefazolin (2 g three times a day) was initiated. A lumbar support brace and short‐term oral morphine treatment were prescribed for pain control. The clinical course was favorable, with rapid regression of the edema and pain and gradual normalization of the CRP level. Cefazolin was discontinued after 10 days of treatment. Given the rapid clinical response, the patient’s age, and the absence of bone or disc involvement on MRI, we opted for an early switch to a 4 week course of oral ciprofloxacin plus close clinical and laboratory monitoring. All the assessments were satisfactory, the inflammatory marker levels continued to fall, and the patient was able to discontinue use of the brace a few days after discharge, with initiation of a structured rehabilitation program.

## 3. Discussion

The interspinous bursa is a synovial structure that can become inflamed in isolation [2]. In children and adolescents, the paravertebral tissue’s rich vascular bed favors the blood dissemination of pyogenic organisms [3]; this may explain the occurrence of infectious bursitis in the absence of associated bone or disc involvement. The combination of (i) short, nonprominent spinous processes, (ii) flexible, nondegenerate interspinous ligaments, and a spine that is not yet affected by degenerative changes means that interspinous bursa does not develop in children and thus explains why interspinous bursitis is exceedingly rare in pediatrics. This presentation contrasts with that of spondylodiscitis, which is more common in children and adolescents [4]. No radiological signs of spondylodiscitis were observed in the present case.

In children, spondylodiscitis represents the primary differential diagnosis due to its higher prevalence [4, 5]. Unlike bursitis, pediatric spondylodiscitis more frequently involves disc and vertebral endplate changes visible on MRI [5]. The absence of bone/disc involvement on MRI, combined with rapid response to targeted antibiotics, strongly favors infectious bursitis over spondylodiscitis in our adolescent patient. The main differential diagnoses for interspinous bursitis are spondylodiscitis, lumbar pyomyositis, and epidural abscess (Table 1) [4]. Furthermore, other rare axial or pelvic infections, such as septic sacroiliitis, should be considered in the broader differential diagnosis of acute inflammatory lower back pain, as they can also present atypically and involve unusual staphylococcal strains [10, 11]. The reference examination is MRI (especially with T1‐ and T2‐weighted sequences) because it can identify an interspinous collection and rule out bone or disc involvement [6, 12]. We can use PET/CT to provide complementary information on the intensity of inflammation and the potential presence of an infectious source or occult endocarditis. The combination of MRI and PET/CT is therefore particularly useful for atypical or early presentations [13].

**TABLE 1:** Key MRI findings differentiating infectious interspinous bursitis from other pediatric spinal infections.

Diagnosis	Key MRI arguments	References
Interspinous Bursitis	Fluid collection or edema—demonstrating T2/STIR hyperintensity and peripheral contrast enhancement—strictly confined to the midline interspinous space; normal anterior column.	Present case; Protat et al. *Joint Bone Spine*. 2023 [6].
Pediatric Spondylodiscitis	Intervertebral disc narrowing, T2 hyperintensity within the disc, and adjacent endplate bone marrow edema.	Principi N, et al. *Int J Mol Sci.* 2016 [7].
Spinal Epidural Abscess	Intraspinal fluid collection (epidural space) with potential thecal sac or spinal cord compression.	Darouiche RO. *N Engl J Med.* 2006 [8].
Paraspinal Pyomyositis	Intramuscular edema (T2/STIR hyperintensity) or ring‐enhancing abscess localized within the paraspinal muscles.	Crum‐Cianflone NF. *Infect Dis Clin North Am.* 2008 [9].

To the best of our knowledge, few literature data on the bacteriological profile of interspinous bursitis have been published (summarized in Table 2). This conservative approach aligns with European pediatric infectious disease guidelines recommending initial IV antibiotics followed by early oral switch for uncomplicated musculoskeletal infections without bone/disc involvement [14]. Our patient’s rapid clinical response (CRP normalization within 10 days) validated this strategy. Treatment is based on a combination of targeted antibiotic therapy, rest, and (in some cases) a lumbar brace for pain relief. The clinical outcome is usually favorable with nonsurgical treatment alone, as observed in our patient [1].

**TABLE 2:** Summary of clinical data, management, and outcomes in pediatric focal septic spinal bursitis.

Reference	Age (years)	Clinical presentation	MRI findings	Microbiological finding	Treatment	Outcome
Protat et al. (2023) [6].	16	Dorsolumbar spine inflammatory pain, fever, inflammatory syndrome	*L1-L2 interspinous septic bursitis erosion of the L1 spinous process*	*Staphylococcus aureus*	Six weeks of antibiotic therapy	Symptoms Resolution
Present case (2026)	16	Lumbar spinal pain, subcutaneous swelling, fever, inflammatory syndrome	*L3-L4 Interspinous collection T1 hypointensity and T2 hyperintensity No associated disc or bone involvement*	Methicillin‐susceptible *Staphylococcus aureus*	IV Cefazolin ‐to‐oral ciprofloxacin & rest	Complete clinical resolution, CRP normalization

Abbreviations: CRP, C‐reactive protein; IV, intravenous.

## Funding

No funding was received for this manuscript.

## Consent

The patient’s parents gave their informed consent to publication of personal medical data and images.

## Conflicts of Interest

The authors declare no conflicts of interest.

## Data Availability

The data that support the findings of this study are available on request from the corresponding author. The data are not publicly available due to privacy or ethical restrictions.
